# Supplementary material for: Towards Zero-Waste Valorization of African Catfish By-Products Through Integrated Biotechnological Processing and Life Cycle Assessment
Source: Gels. 2026 Jan 1;12(1):45. doi: 10.3390/gels12010045 (PMC12841133; doi:10.3390/gels12010045)
Supplement: Supplementary file 1 [file gels-12-00045-s001.zip › gels-4035903-supplementary.pdf]

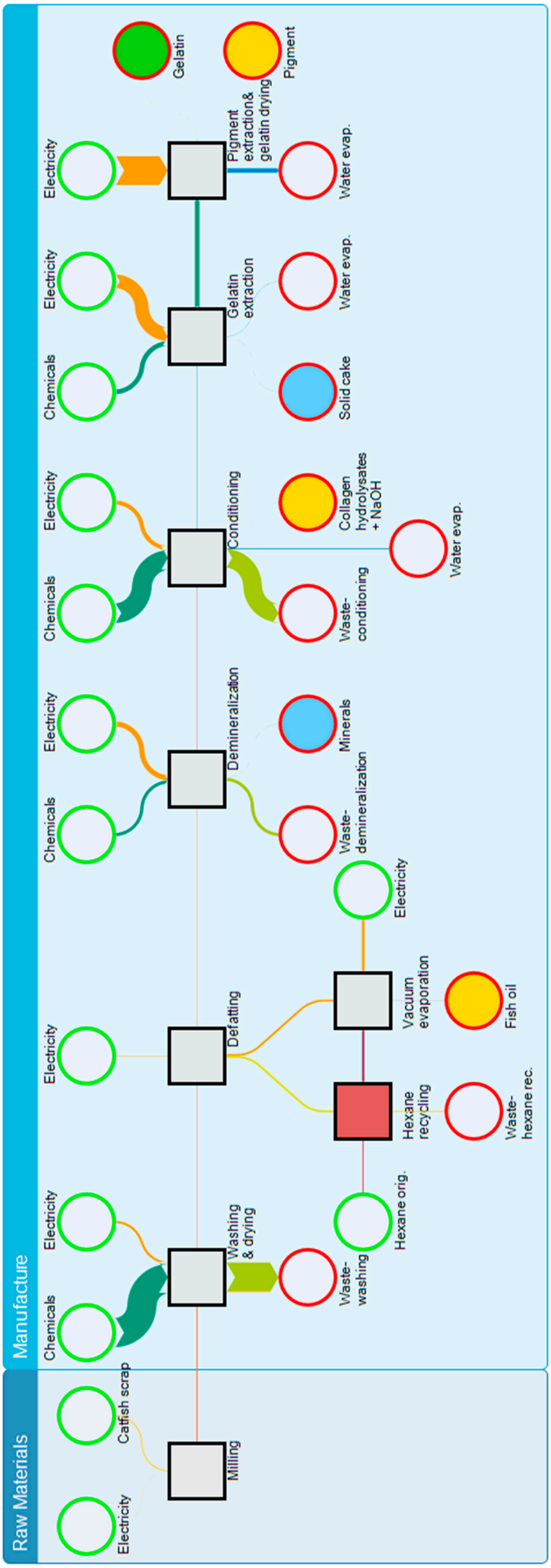

Figure S1. Model of product system containing internal recycling of hexane, Sakey diagram of material and energy flows.

Table S1. Inventory of the product system divided into processes.

Input	Quantity		Output	Quantity	
Milling					
Catfish scrap	33.130	kg	Milled catfish scrap	33.130	kg
Electricity	0.497	kWh			
Washing					
Electricity	22.065	kWh	Washing output	6.096	kg
Milled catfish scrap	33.130	kg	Solid waste	26.835	kg
Sodium hydroxide	0.239	kg	Waste NaCl	2.324	kg
Sodium chloride	2.324	kg	Waste NaOH	0.239	kg
Tap water	1074.411	kg	Waste water	1074.411	kg
Defatting					
Electricity	4.174	kWh	Defatting output	4.950	kg
Hexane mix	71.611	kg	Fat and hexane	72.757	kg
Washing output	6.096	kg			
Demineralization					
Defatting output	4.950	kg	Demineralization output	2.935	kg
Electricity	48.403	kWh	Minerals	2.015	kg
Hydrochloric acid	2.932	kg	Waste HCl	2.932	kg
Tap water	107.839	kg	Waste water	107.839	kg
Conditioning					
Demineralization output	2.935	kg	Collagen hydrolysates	0.288	kg
Electricity	39.600	kWh	Conditioning output	2.665	kg
Sodium hydroxide	0.409	kg	Evaporated water	30.327	kg
Tap water	719.000	kg	Solid NaOH	0.082	kg
			Waste water	689.000	kg
Gelatin extraction					
Conditioning output	2.665	kg	Evaporated water	6.000	kg
Electricity	128.900	kWh	Gelatin and pigment	161.123	kg
Tap water	166.000	kg	Solid cake	1.542	kg
Pigment extraction and gelatin product drying					
Electricity	275.300	kWh	Evaporated water	160.000	kg
Gelatin and pigment	161.123	kg	Gelatin	1.000	kg
			Pigment	0.123	kg
Internal recycling of hexane					
Vacuum evaporation					
Electricity	26.835	kWh	Fish oil	1.146	kg
Fat and hexane	72.757	kg	Hexane after evaporation	71.611	kg
Hexane recycling					
Hexane	27.212	kg	Hexane mix	71.611	kg
Hexane after evaporation	71.611	kg	Hexane waste	27.212	kg

Table S2. Parameters of the fatty acid analysis.

**Gas Chromatography**	Shimadzu GC-2030
Autosampler	AOC-6000
Injection volume	1 µL
Split	1:50
Injection temperature	220 °C
Pressure	43.1 kPa
Total Flow	41.4 mL/min
Column Flow	0.75 mL/min
Purge Flow	3.0 mL/min
Carrier gas	Helium
Flow settings	Linear velocity 32.0 cm/sec
Column	SPB®-PUFA (30 m × 0.25 mm × 0.20 µm)
**Temperature program**	
Oven initial temperature	80 °C (hold 2 min)
Ramp 1	8 °C/min (to 150 °C)
Ramp 2	2 °C/min (to 220 °C)
Final temperature	220 °C
Final Hold	39.25 min
**Mass Spectrometry**	Shimadzu TQ8040 NX
Ion Source	EI 70 eV
Ion Source Temp	200.00 °C
Interface Temp	220 °C
Solvent Cut Time	4.00 min
Detector Gain	0.95 kV
Acquisition mode	Q3 Scan
Start m/z	42.00
End m/z	500.00
Acquisition window	4.05–84.99 min
Scan Speed	10000 u/sec
Event Time	0.050 sec
Identification	NIST20 library matching
